# Dietary Exposure to Excess Saturated Fat During Early Life Alters Hippocampal Gene Expression and Increases Risk for Behavioral Disorders in Adulthood

**DOI:** 10.3389/fnins.2020.527258

**Published:** 2020-09-11

**Authors:** Kathleen C. Page, Endla K. Anday

**Affiliations:** ^1^Department of Biology, Bucknell University, Lewisburg, PA, United States; ^2^College of Medicine, Drexel University, Philadelphia, PA, United States

**Keywords:** maternal diet, high-fat, post-weaning diet, hippocampus, anxiety, depression

## Abstract

**Purpose:**

Maternal and postnatal diets result in long-term changes in offspring brain and behavior; however, the key mediators of these developmental changes are not well-defined. In this study, we investigated the impact of maternal and post-weaning high-fat diets on gene expression of key components mediating hippocampal synaptic efficacy. In addition, we evaluated the risk for impaired stress-coping and anxiety-like behaviors in adult offspring exposed to obesogenic diets during early life.

**Methods:**

Dams were fed a control (C) or high-fat (HF) diet prior to mating, pregnancy, and lactation. Male offspring from control chow and high-fat fed dams were weaned to control chow or HF diets. The forced swim test (FST) and the elevated-plus maze (EPM) were used to detect stress-coping and anxiety-like behavior, respectively. Real-time RT-PCR and ELISA were used to analyze hippocampal expression of genes mediating synaptic function.

**Results:**

Animals fed a HF diet post-weaning spent more time immobile in the FST. Swimming time was reduced in response to both maternal and post-weaning HF diets. Both maternal and post-weaning HF diets contributed to anxiety-like behavior in animals exposed to the EPM. Maternal and post-weaning HF diets were associated with a significant decrease in mRNA and protein expression for hippocampal GDNF, MAP2, SNAP25, and synaptophysin. Hippocampal mRNA expression of key serotonergic and glutamatergic receptors also exhibited differential responses to maternal and post-weaning HF diets. Hippocampal serotonergic receptor 5HT1A mRNA was reduced in response to both the maternal and post-weaning diet, whereas, 5HT2A receptor mRNA expression was increased in response to the maternal HF diet. The glutamate AMPA receptor subunit, GluA1, mRNA expression was significantly reduced in response to both diets, whereas no change was detected in GluA2 subunit mRNA expression.

**Conclusion:**

These data demonstrate that the expression of genes mediating synaptic function are differentially affected by maternal and post-weaning high-fat diets. The post-weaning high-fat diet clearly disturbs both behavior and gene expression. In addition, although the transition to control diet at weaning partially compensates for the adverse effects of the maternal HF diet, the negative consequence of the maternal HF diet is exacerbated by continuing the high-fat diet post-weaning. We present evidence to support the claim that these dietary influences increase the risk for anxiety and impaired stress-coping abilities in adulthood.

## Introduction

The abundance of saturated fats and carbohydrate-dense foods in the Westernized world has contributed to the prevalence of obesity. In fact, epidemiological studies show that in the last two to three decades, more than 50% of women of child-bearing age in the United States are overweight or obese. If the trend continues, more than 80% of adults will be obese by 2030 (Wang et al., 2008). The etiology of obesity is multifactorial and involves complex interactions between genes and the environment. In addition to numerous lifestyle factors which contribute to the development of obesity, a growing body of evidence suggests that genetic programming occurs while still in the womb (Barker et al., 2002).

Obesity in pregnancy represents a significant problem. Not only because of adverse effects on maternal health and pregnancy outcomes such as hypertensive disorders, gestational diabetes, and increased risk for Cesarean section, but also because it poses a risk for the developing fetus (Srinivasan et al., 2006). Childhood obesity has been associated with an increased risk of psychological and behavioral disorders which include depression and anxiety (Rofey et al., 2009), autism spectrum disorder (ASD) (Angelidou et al., 2012), learning deficits and attention deficit disorder (Cserjesi et al., 2007). Moreover, obese adults are 20% more likely to have depressive disorders compared to non-obese subjects (Simon et al., 2006; Waring and Lapane, 2008).

Animal studies have demonstrated that prolonged consumption of a diet high in saturated fat results in increased body weight, hyperinsulinemia, and insulin resistance (Woods et al., 2003). A number of studies have also demonstrated that inflammation, oxidative stress and dysregulation of hormones impair the formation of critical neural circuits in the developing brain (Sullivan et al., 2015). In fact, brain leptin resistance is a well-studied aspect of the obese state. Previous reports indicate that obesity and disturbances in leptin signaling markedly affect the expression of neuronal and glial proteins and that leptin binding to its receptor, which is expressed at a remarkably high level in the hippocampus (Caron et al., 2010), is required for neurogenesis and efficient synaptic transmission (Grillo et al., 2011; McGregor and Harvey, 2018).

It has also been shown that excess dietary fat consumption may contribute to a physiological background of chronic stress since elevations in the level of adrenocorticotropic hormone and glucocorticoid are associated with high-fat feeding (Tannenbaum et al., 1997). Stress results in loss of neurons and glia in the hippocampus (Duman and Duman, 2015), and the adverse effects of glucocorticoids on hippocampal spine synapses and neurogenesis are well-documented (McEwen et al., 2016). In addition, the hippocampus is an integral part of neural circuits regulating the HPA axis response to stress. Disruption of HPA axis-controlled glucocorticoid release is a common feature of depression (Duman, 2014a) and a heightened HPA-axis response to stress accompanies high-fat feeding (Dutheil et al., 2016). It is becoming clear that HPA axis hyperactivity and excess circulating glucocorticoid in response to a HF diet may lead to altered stress responsiveness and behavioral alterations such as mood disorders.

The claim that a HF diet disrupts HPA axis regulation (Winther et al., 2018) is further supported by studies from de Kloet’s laboratory. Their results demonstrate that the capacity for an individual to cope with stress is coordinated via glucocorticoid action on the mineralcorticoid receptor (MR) network, particularly in the hippocampus. In fact, subsequent management of stress adaptation via the low affinity glucocorticoid receptor (GR) is dependent on the coordinated regulation of the MR:GR ratio where MR is the critical mediator of an animal’s resilience to stress (de Kloet et al., 2016). Previous results from our lab showed that a reduction in hippocampal MR but not GR expression was associated with early life exposure to excess glucocorticoids, and that this change in the MR:GR ratio was associated with an increase in hippocampal drive and HPA axis hyperactivity (Shoener et al., 2006).

We have now turned our attention to the impact of maternal and post-weaning HF diets on the expression of genes mediating hippocampal neurogenesis and synaptic efficacy, functions that impact an individual’s emotional balance. Neural plasticity and experience-dependent plasticity are particularly vulnerable to gene–environment interactions during developmental periods such as early life, and adverse conditions during these times could induce changes that increase the risk of psychopathologies such as anxiety and depression in adulthood. Although perturbations in each individual gene susceptible to gene–environment interactions contribute a small fraction to the total risk for behavioral disorders, it is likely that overlapping sets of susceptibility genes are at play. This makes the timing and dose-response outcome difficult to discern (Lopizzo et al., 2015). In order to explore the timing and extent of HF diet exposure, we chose to investigate the effects of a diet high in saturated fat during two distinct developmental times; (1) the period between gestation and the end of lactation which reflects the maternal diet and (2) the period from weaning to adulthood which constitutes the post-weaning diet. Each diet period includes environmental variables that may impact genetic programming differentially, particularly in the developing brain. We hypothesized that maternal and post-weaning diets high in saturated fat exert deleterious effects on the expression of hippocampal genes mediating synaptic plasticity and increase the propensity for behavioral disorders in adulthood. To investigate this possibility, we employed the forced swim test (FST) and the elevated-plus maze (EPM) to assess alterations in stress-coping and anxiety-like behavior, respectively.

We also examined expression data for proteins mediating hippocampal plasticity, synaptic function, and associated behavioral manifestations. We hypothesized that expression of these key genes is perturbed in response to the consumption of a high-fat diet. We analyzed the mRNA and protein expression for GDNF, a neurotrophin required for survival and efficacy of neuronal contacts, and for MAP2, a protein which reflects the capacity for receiving synaptic inputs along dendritic outgrowths. In addition, the expression of synaptosomal-associated protein 25 (SNAP25) and synaptophysin was measured since these two proteins are critical for presynaptic vesicle transport and fusion. The mRNA levels for the key post-synaptic serotonin receptors, 5HT1A and 5HT2A, and critical subunits of the glutamate responsive AMPA receptor, GluA1 and GluA2, were also evaluated. The choice of molecular markers was based on literature focused on synaptic dysfunctions that underpin an individual’s risk for emotional imbalance, particularly anxiety and depression (Duman and Duman, 2015; McEwen et al., 2016; Tsybko et al., 2017). To date, studies designed to explore the effects of high-fat diets on anxiety and stress-coping strategies are not conclusive; however, these studies indicate that disturbances in the integrity and/or functionality of the hippocampus are involved in the etiology of mood disorders.

## Materials and Methods

### Animals

Thirty-two Sprague-Dawley dams (Hilltop Laboratories) were split into two nutritional groups as described previously (35). Sixteen of the dams were placed on standard rat chow providing 3.85 kcal/g dry wt, 20 kcal% protein, 70 kcal% carbohydrate, and 10 kcal% saturated fat (D12450H; Research Diets, New Brunswick, NJ, United States). The remaining 16 dams were fed a high-fat diet providing 4.73 kcal/g dry wt, 20 kcal% protein, 35 kcal% carbohydrate, and 45 kcal% saturated fat, mostly in the form of lard (D12451; Research Diets), for 1 month before mating. Standard and high-fat diets were sucrose matched. Pregnant dams were housed singly and continued on their specified diets. At birth, litters were culled to 10 offspring per dam to standardize lactation demands. At weaning, four male offspring were chosen at random from each of the 16 control dams and split into two nutritional groups: two male offspring from each of the 16 control dams (C) were weaned to standard chow (C) diet (CC, *n* = 16), and the other two offspring from each control dam (C) were weaned to a 45 kcal% saturated high-fat (HF) diet (CHF, *n* = 16). Four male offspring from each dam fed a HF diet were also split into two nutritional groups at weaning: two of the four offspring from each of the 16 HF fed dams (HF) were weaned to standard chow (C) diet (HFC, *n* = 16), and the remaining two male offspring from each HF fed dam (HF) were weaned to the high-fat (F) diet (HFF; *n* = 16). Results obtained from each pair of male offspring taken from a given dam were averaged and represented as *n* = 1. The final sample size was *n* = 16 for each group.

All offspring were continued on their respective diets into adulthood. At 126 to 130 days of age, adult males from each experimental treatment were separated by litter (*n* = 16) and tested for anxiety-like behavior using the EPM. Since it has been shown that anxiogenic effects remain even after a 1-week interval between behavioral tests (Andreatini and Bacellar, 1999), our animals were allowed to acclimate and return to baseline for 10 days prior to testing the animals at 136 to 140 days for depressive-like behavior using the FST.

### Behavioral Testing: Forced Swim Test and Elevated-Plus Maze

The FST was used to assess the response to acute inescapable stress which reflects the activity of neuronal networks that may be impaired in depression, ASD, and other disorders (Commons et al., 2017). More specifically, the increased immobility time during forced swim was used in our study as a measure for stress-coping behavior in rodents. In this study we used a repeated-trial procedure similar to the original FST protocol (Porsolt et al., 1977). Since rats remain immobile for a higher proportion of time in the second trial, this approach provides a more suitable baseline for measuring a decrease or increase in immobility duration. An interval of 24 h between the pre-test and the test trial was used. On day one, rats were individually immersed in a water-filled (23–25°C) plastic cylinder (46 cm high × 20 cm diameter) filled with tap water to 30 cm water depth which prevents them from touching the bottom. The animals were exposed to the forced swim for 15 min. After 24 h, the test trial was conducted similarly, however, in this trial animals were subjected to the forced swim for only 5 min and time spent immobile during each one min interval over the 5-min period was recorded along with swimming activity. All sessions were videotaped and recorded for analysis using ANY-MAZE video-tracking system and time spent immobile, swimming, or climbing for the four treatment groups was analyzed using a two-way ANOVA [maternal diet X post-weaning diet].

General exploratory and anxiety-like behaviors were evaluated using EPM apparatus consisting of a black acrylic cross with a shared central square (12 cm × 12 cm) with two opposite open arms and two opposite closed arms (50 cm × 12 cm). The closed arms were enclosed by 50-cm high walls. The test rat was placed on the central square facing a corner which allowed equal choice of entering an open or closed arm. The animal explored the apparatus during a 5 min session. The behavior was recorded using ANY-MAZE tracking system. Time spent and distance traveled in the open and closed arms were quantified. At the end of each session, the number of fecal boli were counted and the apparatus was thoroughly cleaned.

Upon completion of behavior testing the animals were maintained in their specific groups for 10–14 days prior to sacrifice to ensure that all animals had returned to a stable physiological baseline after behavioral testing. At 150 days of age, individual animals from each group were decapitated in a staggered fashion (CC, CHF, HFC, HFF, repeat). Immediately upon death, trunk blood was drained into serum collection tubes containing gel activator, whole hippocampi were rapidly dissected from each side of the brain and rapidly frozen using liquid nitrogen. Body weight was recorded and visceral fat pads, both retroperitoneal and gonadal, were excised and weighed.

### Collection of Hippocampi

Hippocampi from each animal were rapidly dissected and frozen in liquid nitrogen. This was carried out during the morning phase of the circadian cycle (between 800 and 1000 h) when each animal was 150 days of age. These studies were conducted in accordance with the NIH Guide for Care and Use of Laboratory Animals, and all animal procedures have been reviewed and approved by the Animal Care and Use Committee of Bucknell University.

### RNA Isolation and RT-PCR

Total RNA was isolated from each hippocampus using TRIzol (Invitrogen, Carlsbad, CA, United States) according to the manufacturer’s instructions. Purified total RNA was reverse transcribed using RETROscript (Ambion, Austin, TX, United States) in accordance with the manufacturer’s recommended procedure. Real-time PCR was performed on an iCycler iQ Real Time PCR Detection System (Bio-Rad, Hercules, CA, United States) using SYBR Green Supermix (Bio-Rad, Hercules, CA, United States). Target genes were amplified using the thermocycling program and controls previously described (Page et al., 2014). Primers were constructed using the NCBI online database^1^ and sequence specificity of each primer pair (Table 1) was confirmed using BLAST^2^. All primers were ordered from MWG Oligo Synthesis (High Point, NC, United States). Efficiency of the primers was calculated, and gel electrophoresis was conducted on primers to exclude those with DNA contamination. In each sample, the gene of interest was co-amplified with a standard housekeeping gene 18S ribosomal RNA. Before the experimental plates were analyzed, real-time PCR control plates were run for each primer pair: double-distilled H_2_O was substituted for cDNA to verify that exogenous DNA was not present and 2 μg of isolated total RNA was substituted for cDNA in the PCR reaction to rule out genomic DNA contaminants. Both negative controls showed no amplification even after 35 cycles. The following genes were amplified from hippocampal-derived cDNA: glial-derived neurotrophic factor (GDNF), microtubule-associated protein 2 (MAP2), synaptosomal-associated protein (SNAP25), synaptophysin, the ionotropic glutamate AMPA receptor subunits, GluA1 and GluA2, and the G-protein coupled serotonin receptors, 5HT1A and 5HT2A. In each sample, the gene of interest was co-amplified with the standard housekeeping gene, 18S ribosomal RNA, to control for potential differences in pipette load volume.

**TABLE 1:** Primer sequences (*GenBank/NCBI*) used in real-time PCR.

Primer ID	Primer sequence 5′–3′	Accession no.	Length, nt
18S	Fwd: AAACGGCTACCACATCCAAG Rev: GGCCTCGAAAGAGTCCTGTA	M11188	76
GDNF	Fwd: GGAGACCGGATCCGAGGTG Rev: GCGCTTCGAGAAGCCTCTTA	NM_019139	136
GluA1	Fwd: CGACCCTCCCCCGGAACAGT Rev: GGCTGACCACCCGGCCATTC	NM_031608	75
GluA2	Fwd: TGTGGAGCCAAGGACTCGGGA Rev: TTGCCAAACCAAGGCCCCCG	NM_017261	103
MAP2	Fwd: CCCTCCTCGCAGGGGCGTAT Rev: TCTGACCTGGTGGTCCGTCGT	NM_2013066	93
SNAP-25	Fwd: CCTTCCCTCCCTACCCGGCG Rev: CGGGGCCAGCAAGTCAGTGG	NM_030991	115
SYP	Fwd: GAATCAGCTGGTGGCTGGGGG Rev: CACGCTCAGCCGAAGCTCCC	NM_012664	136
5HT1A	Fwd: TGTTGCTCATGCTGGTTCTC Rev: CCGACGAAGTTCCTAAGCTG	NM_000524	88
5HT2A	Fwd: CTGCTGGGTTTCCTTGTCAT Rev: ATCCAGATCGCACAGAGCTT	NM_017254	106

*Final concentration of each primer was 1 μM. Nucelotides is abbreviated nt. GDNF, glial cell-derived neurotrophic factor; GluA1 and GluA2, AMPA receptor subunits; MAP2, microtutule-associated protein2; SNAP-25, synaptosomal nerve-associated protein 25; Syp, synaptophysin; 5HT1A and 5HT2A, serotonin receptors.*

### Data Analysis for Quantitative qPCR

The cycle numbers at which amplified DNA samples exceeded a computer-generated fluorescence threshold level (C_*T*_) were normalized and compared to determine relative gene expression. There were no statistical differences in 18S C_*T*_ values across different groups (data not shown). Higher cycle number values indicate lower initial concentrations of cDNA, and thus lower levels of mRNA expression. Each sample was run in triplicate, and averaged triplicates were used to assign cycle threshold (C_*T*_) values. dC_*T*_ values were generated by subtracting experimental C_*T*_ values from the C_*T*_ values for 18S targets co-amplified with each sample. The mean dC_*T*_ value per amplified gene target for the CC (control) group was subtracted from itself to set the difference (ddC_*T*_) to 0 and the 2^*ddCT*^ to 1. The mean dC_*T*_ values for each of the other three groups were then subtracted from the dC_*T*_ for the CC group such that the difference in values (ddC_*T*_) would be set relative to the CC group. The ddC_*T*_ values were then calculated as powers of 2 (2^*ddCT*^) to account for the exponential doubling of the polymerase chain reaction and the CC group with its ddC_*T*_ set to 0 would always have its 2^*ddCT*^ equal to one arbitrary unit.

### Protein Levels Determined for Hippocampal GDNF, MAP2, SNAP25, and Synaptophysin Using ELISA

Rapidly dissected whole hippocampi were immediately frozen using liquid nitrogen and kept at 80°C until use. Tissue lysates for ELISA analysis were prepared according to the manufacturer’s protocol (My Biosource, San Diego, CA, United States). Ice-cold homogenate was centrifuged (15 min at 10,000 *g*, 4°C), and supernatant was removed. Protein concentrations of the supernatant were quantified using the Bradford method. Supernatant protein concentration for the gene products of interest, GDNF, SNAP25, MAP2 or Synaptophysin, were analyzed using target specific ELISA kits according to manufacturer’s instructions (My Biosource). Target protein concentration (pg/ml) was normalized using total protein concentration of the supernatant (pg target/mg protein). Protein determinations were made using the Bradford assay.

### Statistical Analysis

Gene expression and data from the FST and EPM were analyzed using two-way ANOVA of maternal diet X post-weaning diet (maternal diet effect CC vs. HFC; CHF vs. HFF and post-weaning diet effect CC vs. CHF; HFC vs. HFF). Multiple comparisons were made using Bonferroni *post hoc* analysis (SPSS, Statistical Analysis Software, Chicago, IL, United States). Statistical significance was confirmed if *P* <0.05.

## Results

### Effects of Excess Saturated Fat Consumption on Behavior

We have established a metabolic profile for rats consuming high fat diets and reported in a previous study that animals exposed to excess saturated fat during early development exhibit significant increases in body weight, energy intake, adipose deposition, and higher circulating levels of leptin, insulin, and glucocorticoid (Page et al., 2009). Our current study was designed to investigate whether exposure to excess saturated fat during development would influence adult behaviors such as stress-coping or anxiety. Using the FST, we measured the behavioral response of an animal to an acute inescapable stressor. Results from this stress-coping test can be used to detect disturbances in neural networks that underpin the development of anxiety, depression, and ASD (Commons et al., 2017).

**FIGURE 1:**
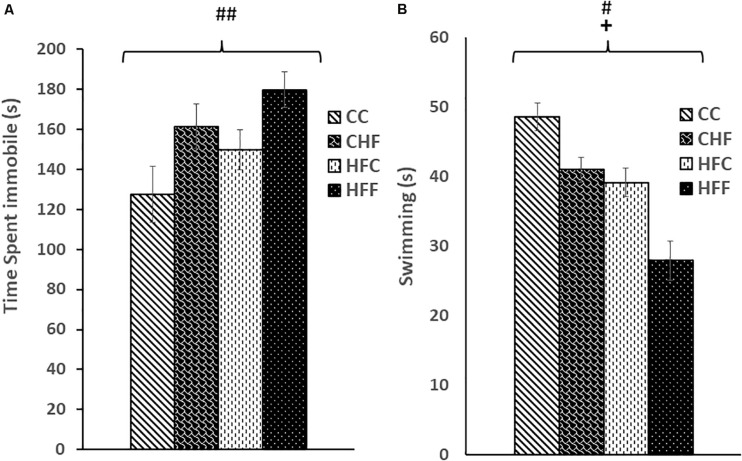
Forced Swim Test performance was measured in adult male rat offspring from chow-fed dams or high-fat fed dams fed either chow or high-fat food post-weaning: control offspring fed chow (CC), control offspring fed high-fat food (CHF), high-fat offspring fed chow (HFC), and high-fat offspring fed high-fat food (HFF). **(A)** Total immobility increased significantly in response to the post-weaning high-fat diet (post-weaning diet effect: *F*_(3,61)_ = 6.58, *P* = 0.01). Increased immobility in response to a maternal high-fat diet did not reach significance (maternal diet effect: *F*_(3,61)_ = 4.15, *P* = 0.058). **(B)** Swim time was reduced by both the maternal (maternal diet effect: *F*_(3,61)_ = 4.36, *P* = 0.038) and post-weaning high-fat diet (post-weaning diet effect: *F*_(3,61)_ = 5.61, *P* = 0.024). No significant interactions were detected using two-way ANOVA. Values are means +SEM where *n* = 16 for each group. **^+^***P* <0.05 (significant maternal diet effect); **^#^***P* <0.05, **^##^***P* <0.01 (significant post-weaning diet effect).

Using the FST, we found that excess saturated fat content in the post-weaning diet markedly increased the amount of time an animal spent immobile [post-weaning diet effect; *F*_(3,61)_ = 6.58, *P* = 0.01; CC vs. CHF, HFC vs. HFF] (Figure 1A). Moreover, the amount of time spent immobile following exposure to the maternal HF diet increased but did not reach significance [maternal diet effect; *F*_(3,61)_ = 4.15, *P* = 0.058; CC vs. HFC, CHF vs. HFF] (Figure 1A). In contrast, time spent swimming was significantly decreased in response to both the maternal diet (*F*_(3,61)_ = 4.36, *P* = 0.038) and post-weaning diet (*F*_(3,61)_ = 5.61, *P* = 0.024) (Figure 1B).

**FIGURE 2:**
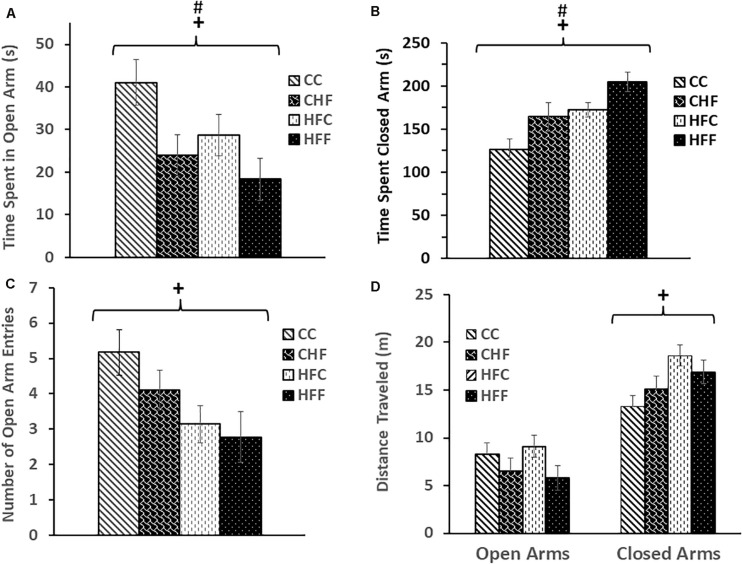
Elevated-plus maze was used to evaluate anxiety-like behavior in adult male rat offspring: CC, CHF, HFC, or HFF. **(A)** Time spent in the open arms was significantly reduced in response to both the maternal (maternal diet effect: *F*_(3,61)_ = 5.17, *P* = 0.034) and post-weaning high-fat diet (post-weaning diet effect: *F*_(3,32)_ = 4.36, *P* = 0.049); whereas, **(B)** time spent in the closed arms was increased in response to both the maternal (maternal diet effect: *F*_(3,61)_ = 6.48, *P* = 0.016) and post-weaning high-fat diet (post-weaning diet effect: *F*_(3,61)_ = 5.52, *P* = 0.026). **(C)** The number of open-arm entries was significantly reduced in response to the maternal high-fat diet (maternal diet effect: *F*_(3,61)_ = 4.19, *P* = 0.042) and **(D)** the maternal diet was associated with an increase in distance traveled by adult male offspring in the closed arms (maternal diet effect: *F*_(3,61)_ = 5.34, *P* = 0.027). No significant interactions were detected using two-way ANOVA. Values are means +SEM (*n* = 16 for each group). **^+^***P* <0.05 (significant maternal diet effect); **^#^***P* <0.05 (significant post-weaning diet effect).

Behavioral changes were also observed during EPM testing. Time spent in the open arms was significantly decreased in response to both the maternal diet (*F*_(3,61)_ = 5.17, *P* = 0.034) and post-weaning diet (*F*_(3,61)_ = 4.36, *P* = 0.049) (Figure 2A), whereas, time spent in the closed arms was significantly increased in response to the maternal (*F*_(3,61)_ = 6.48, *P* = 0.016) and post-weaning HF diet (*F*_(3,61)_ = 5.52, *P* = 0.026) (Figure 2B). Interestingly, a reduction in the number of open arm entries (*F*_(3,61)_ = 4.19, *P* = 0.042) (Figure 2C) and an increase in the distance traveled in the closed arms (*F*_(3,61)_ = 5.34, *P* = 0.027) (Figure 2D) was significant only in response to excess saturated fat in the maternal diet.

### Effects of Excess Saturated Fat on Hippocampal GDNF and MAP2 mRNA and Protein Expressions

**FIGURE 3:**
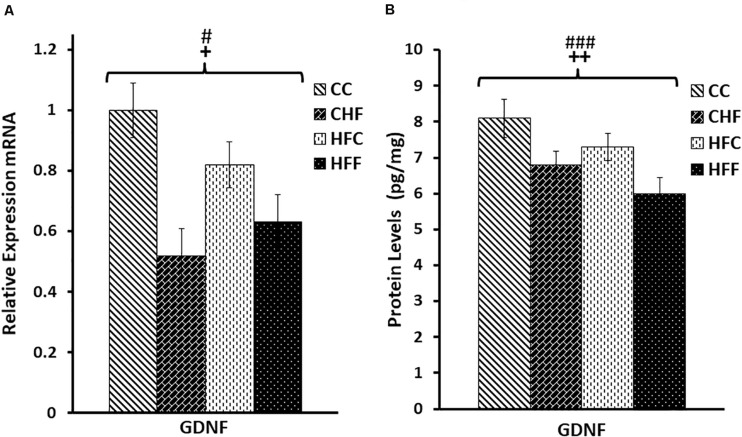
Relative levels of GDNF mRNA and protein expression in the hippocampus. **(A)** Using quantitative real-time PCR (qRT-PCR), glial cell-derived neurotrophic factor (GDNF) mRNA expression was determined to be significantly reduced in response to both the maternal (*F*_(3,57)_ = 3.92, *P* = 0.049) and post-weaning HF diet (*F*_(3,57)_ = 4.87, *P* = 0.034). Values are expressed in relative units where the CC group is set to 1. **(B)** Significant reductions in GDNF protein levels (pg/mg) were also associated with both the maternal (*F*_(3,57)_ = 8.65, *P* = 0.004) and the post-weaning HF diet (*F*_(3,57)_ = 11.64, *P* = 0.001) using ELISA. No significant interactions were detected using two-way ANOVA. Values are means +SEM (*n* = 16 for each group). **^+^***P* <0.05, **^++^***P* <0.01 (significant maternal diet effect); **^#^***P* <0.05, **^###^***P* <0.001 (significant post-weaning diet effect).

To evaluate the effects of HF diets on brain development, more specifically hippocampal neurogenesis, we measured the expression of GDNF, a key neurotrophic factor, as well as MAP2, a major dendritic marker. Two-way ANOVA (maternal diet X post-weaning diet) revealed a significant reduction of hippocampal GDNF mRNA (Figure 3A) in response to both the maternal (*F*_(3,57)_ = 3.92, *P* = 0.049) and post-weaning high-fat diet (*F*_(3,57)_ = 4.87, *P* = 0.034), although the maternal diet effect is mainly due to the significant difference between CC and HFC animals. Moreover, both the maternal (*F*_(3,57)_ = 8.65, *P* = 0.004) and post-weaning diet (*F*_(3,57)_ = 11.64, *P* = 0.001) contributed to the reduction in GDNF protein expression in an independent fashion since no interactions were detected (Figure 3B).

**FIGURE 4:**
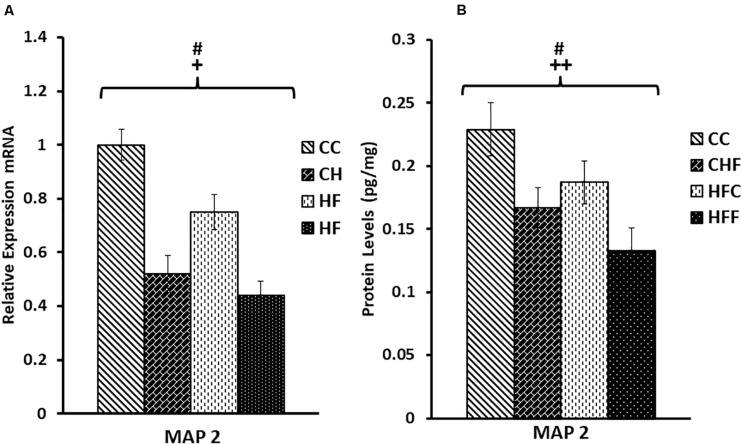
Relative levels of MAP2 mRNA and protein expression in the hippocampus. **(A)** Using qRT-PCR, hippocampal microtubule-associated protein 2 (MAP2) mRNA expression was significantly reduced in response to the maternal diet (*F*_(3,57)_ = 3.28, *P* = 0.05) and post-weaning high-fat diets (*F*_(3,57)_ = 6.76, *P* = 0.013). Values are expressed in relative units where the CC group is set to 1. **(B)** Significant reductions in MAP2 protein levels (pg/mg) were also detected in response to both the maternal (*F*_(3,57)_ = 9.28, *P* = 0.004) and post-weaning HF diets (*F*_(3,57)_ = 5.67, *P* = 0.023) using ELISA. No significant interactions were detected using two-way ANOVA. Values are means +SEM (*n* = 16 for each group). **^+^***P* <0.05,**^++^***P* <0.01 (significant maternal diet effect); **^#^***P* <0.05 (significant post-weaning diet effect).

A similar expression pattern was detected for MAP2 (Figure 4). Again, MAP2 mRNA level was significantly reduced in response to both the maternal (*F*_(3,57)_ = 3.28, *P* = 0.05) and post-weaning diet (*F*_(3,57)_ = 6.76, *P* = 0.013). Moreover, both maternal (*F*_(3,57)_ = 9.28, *P* = 0.004) and post-weaning (*F*_(3,57)_ = 5.67, *P* = 0.023) high-fat diets contributed to the reduction in MAP2 protein expression. No significant interactions were detected using two-way ANOVA.

### Effects of Excess Saturated Fat Consumption on mRNA and Protein Expression of Hippocampal Genes Mediating Synaptic Vesicle Release

**FIGURE 5:**
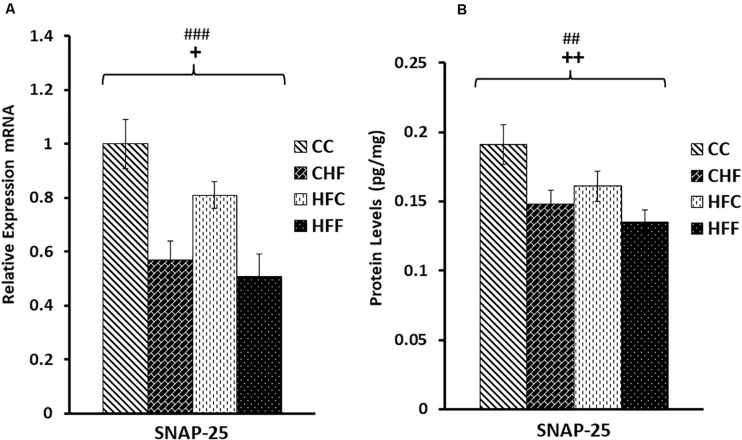
Relative levels of SNAP25 mRNA and protein expression in the hippocampus. **(A)** Using qRT-PCR, hippocampal synaptosomal-associated protein 25 (SNAP25) mRNA expression was significantly reduced in response to both maternal (*F*_(3,57)_ = 5.11, *P* = 0.038) and post-weaning high-fat diets (post-weaning diet effect; *F*_(3,57)_ = 12.71, *P* = 0.001). Values are expressed in relative units where CC group is set to 1. **(B)** Significant reduction in SNAP25 protein levels (pg/mg) was also detected in response to both the maternal (*F*_(3,57)_ = 8.36, *P* = 0.007) and post-weaning HF diets (*F*_(3,57)_ = 10.53, *P* = 0.003) using ELISA. No significant interactions were detected using two-way ANOVA. Values are means +SEM (*n* = 16 for each group). **^+^***P* <0.05,**^++^***P* <0.01 (significant maternal diet effect); **^##^***P* <0.01, **^###^***P* <0.001 (significant post-weaning diet effect).

**FIGURE 6:**
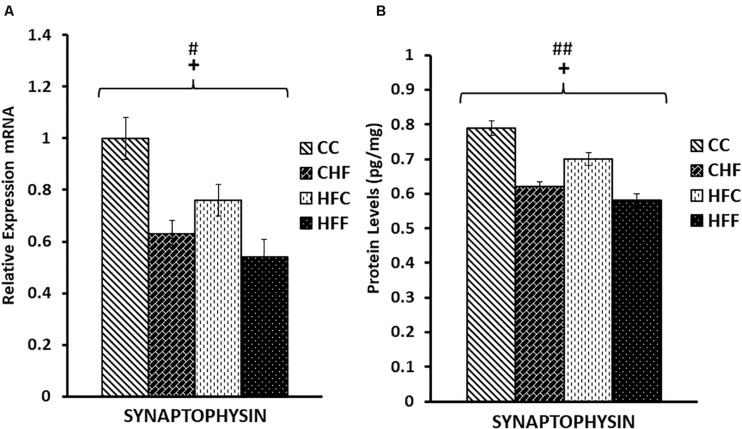
Relative levels of Synaptophysin mRNA and protein expression in the hippocampus. **(A)** Using real-time PCR, hippocampal synaptophysin mRNA expression was significantly reduced in response to both maternal (*F*_(3,39)_ = 4.14, *P* = 0.038) and post-weaning high-fat diets (post-weaning diet effect; *F*_(3,39)_ = 7.47, *P* = 0.014). Values are expressed in relative units where CC group is set to 1. **(B)** Significant reductions in synaptophysin protein levels (pg/mg) were also detected in response to both the maternal (*F*_(3,39)_ = 6.65, *P* = 0.032) and post-weaning HF diets (*F*_(3,39)_ = 8.64, *P* = 0.004) using ELISA. No significant interactions were detected using two-way ANOVA. Values are means +SEM (*n* = 16 for each group). **^+^***P* <0.05 (significant maternal diet effect); **^#^***P* <0.05, **^##^***P* <0.01 (significant post-weaning diet effect).

Neuronal function is exquisitely dependent on synaptic vesicle release. To determine the effects of HF diets on synaptic function, we measured the expression of two key genes mediating synaptic vesicle release, SNAP25 and synaptophysin. Expression of SNAP25 mRNA was reduced in response to both the maternal (*F*_(3,57)_ = 5.11, *P* = 0.038) and post-weaning high-fat diets (*F*_(3,57)_ = 12.71, *P* = 0.001) (Figure 5A). This decrease was also translated into a significant reduction in SNAP25 protein levels (pg/mg) in response to both maternal (*F*_(3,57)_ = 8.36, *P* = 0.007) and post-weaning (*F*_(3,57)_ = 10.53, *P* = 0.003) high-fat diets (Figure 5B). This pattern of reduced expression was once again detected in synaptophysin mRNA [maternal diet effect, *F*_(3,57)_ = 4.14, *P* = 0.038; post-weaning diet effect; *F*_(3,57)_ = 7.47, *P* = 0.014] (Figure 6A) and protein levels [maternal diet effect, *F*_(3,57)_ = 6.65, *P* = 0.032; post-weaning diet effect; *F*_(3,57)_ = 8.64, *P* = 0.004] (Figure 6B).

### Effects of Excess Saturated Fat Consumption on the mRNA Expression of Genes Mediating Serotonergic and Glutamatergic Function in the Hippocampus

**FIGURE 7:**
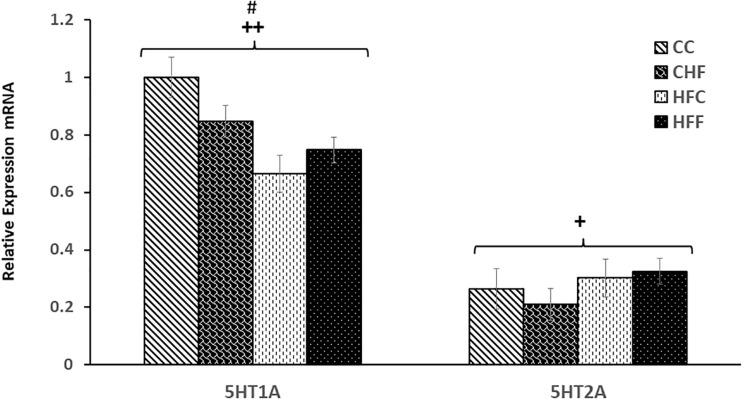
Relative levels of serotonin receptor expression in the Hippocampus. Hippocampal serotonin receptor, 5HT1A and 5HT2A, mRNA expression in the four treatment groups (CC, CHF, HFC, HFF) was measured using qRT-PCR analysis. Values are expressed in relative units where the CC group is set to 1. Serotonin receptor 1A (5HT1A) mRNA expression was significantly reduced in response to both maternal diet (5HT1A, *F*_(3,39)_ = 7.13; *P* = 0.007) and post-weaning diet (*F*_(3,39)_ = 6.49; *P* = 0.027). In contrast, an increase in 5HT2A receptor was detected only in response to the maternal diet (*F*_(3,39)_ = 7.28; *P* = 0.039). No significant interaction was detected between maternal and post-weaning diet using two-way ANOVA. Values are means +SEM (*n* = 16 for each group). **^+^***P* <0.05,**^++^***P* <0.01 (significant maternal diet effect); **^#^***P* <0.05 (significant post-weaning diet effect).

**FIGURE 8:**
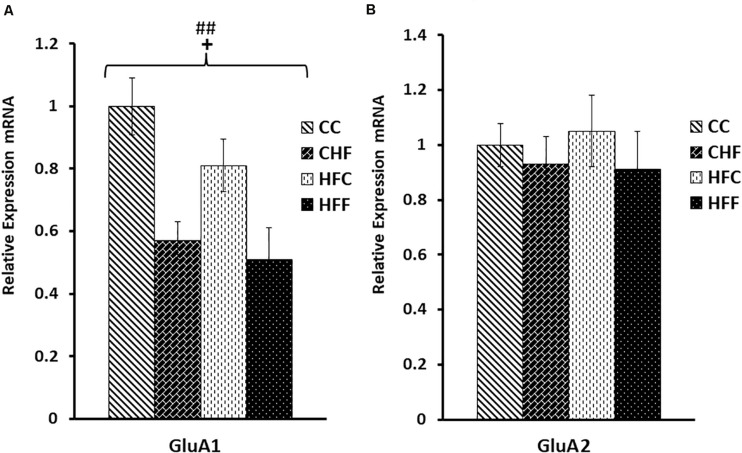
Glutamate AMPA receptor subunit mRNA expression in the hippocampus. Hippocampal mRNA expression for the glutamate AMPA receptor subunits, GluA1 and GluA2, was measured in the CC, CHF, HFC, and HFF groups using qRT-PCR. Values are expressed in relative units where the CC group is set to 1. **(A)** GluA1 subunit expression was significantly reduced in response to both the maternal (*F*_(3,39)_ = 4.06, *P* = 0.047) and post-weaning HF diet (*F*_(3,39)_ = 8.04, *P* = 0.008). **(B)** No significant changes were measured for hippocampal GluA2 mRNA expression using two-way ANOVA. Values are means +SEM (*n* = 16 for each group). **^+^***P* <0.05 (significant maternal diet effect); **^##^***P* <0.01 (significant post-weaning diet effect).

Post-synaptic receptors are also key to synaptic efficacy and plasticity. To assess the status of key post-synaptic receptors following nutritional manipulation, we examined the hippocampal mRNA expression of important serotonin receptor subtypes, 5HT1A and 5HT2A, as well as two key subunits of the glutamate AMPA receptor, GluA1 and GluA2. Serotonin receptor 1A (5HT1A) mRNA was significantly decreased in response to both the maternal (*F*_(3,57)_ = 7.13; *P* <0.01) and post-weaning diet (*F*_(3,57)_ = 6.49; *P* <0.01) (Figure 7), whereas the 5HT2A receptor mRNA expression was significantly increased only in offspring from dams fed a diet high in saturated fat [maternal effect: (*F*_(3,57)_ = 7.28, *P* <0.05)] (Figure 7). The mRNA expression of key subunits composing the glutamate-gated AMPA receptor was also differentially affected by nutritional manipulation. GluA1 mRNA level was significantly reduced by both the maternal (*F*_(3,57)_ = 4.06; *P* <0.05) and post-weaning diet (*F*_(3,57)_ = 8.04, *P* <0.01) (Figure 8A), whereas, the expression of the GluA2 subunit was not significantly changed (Figure 8B).

## Discussion

Results from this study demonstrate that exposure to excess dietary fat during development adversely affects the expression of genes mediating hippocampal neurogenesis and plasticity and leads to emotional imbalance. Previous studies have shown that obesity is associated with both anxiety- (Mizunoya et al., 2013; Ogrodnik et al., 2019) and depressive-like behavior (Hryhorczuk et al., 2013), and that the negative impact on hippocampal neurogenesis in depression model rats can be alleviated by the antidepressant, clomipramine (Liu et al., 2008). In our study, the expression of genes critical to neurogenesis and plasticity were, on average, significantly reduced in response to HF diet exposure, and behaviors reflecting the animals emotional state were disturbed.

We previously investigated the impact of HF diets on key metabolic factors which showed that maternal and post-weaning HF diets are associated with significantly higher body weight and visceral fat mass as well as markedly increased serum levels of leptin, insulin, and glucocorticoid (Page et al., 2009). These characteristics are indicative of the obese phenotype that is often accompanied by neuroinflammation and oxidative stress (Edlow, 2017), two stressors implicated in the development of anxiety and depression (Patki et al., 2013).

In our current study, we evaluated the effect of a HF diet on an animal’s stress coping capacity or anxiety-like response using the FST and EPM, respectively. Results from the FST indicated that adult male offspring consuming excess saturated fat post-weaning spent significantly more time immobile. The increase in time spent immobile in animals exposed to a maternal HF diet was also detected, but this change did not reach significance. The CHF and HFF adult offspring were most susceptible to disruption in their stress coping strategies. Although depressive tendencies are not directly measured using the FST, this behavioral test does provide information with regard to an animal’s ability to cope with an inescapable stressor (Molendijk and de Kloet, 2019), and changes in stress responsiveness are associated with an animal’s vulnerability to depression (de Kloet et al., 2016). In fact, the neural networks that control coping strategies in response to acute stress overlap heavily with those affected by depression (Commons et al., 2017). For example, responses to acute stress are under the control of the glucocorticoid-regulated HPA axis, and dysregulation of this neuroendocrine feedback loop often accompanies depressive illness (Gold et al., 2015).

In our previous study, we found a significant increase in circulating glucocorticoid in animals exposed to excess saturated fat *in utero* and/or during the post-weaning period. This suggests that HPA axis feedback regulation has been disturbed. In fact, using the synthetic glucocorticoid, dexamethasone, we demonstrated that excess glucocorticoid exposure during the prenatal period increases hippocampal drive on the HPA axis in adulthood (Shoener et al., 2006). It is likely that the resulting HPA hyperactivity predisposes the animal to persistent physiological changes such as disturbances in the stress response and susceptibility to stress-induced anxiety and depression. In fact, rats or mice exposed to chronic HF diets develop various impairments, including inflammation, high corticosterone (CORT) levels, neuronal atrophy, decreased neurogenesis, and decreased neurotrophic factor expression in regions such as the hippocampus where glucocorticoid receptors are particularly abundant (Dutheil et al., 2016). These disturbances most likely contribute to the impaired stress coping measured in our HF fed animals.

In addition to the diminished stress coping strategy detected in our experimental animals using the FST, results from our EPM testing demonstrate that maternal and post-weaning HF diets are also associated with a significant increase in anxiety-like behavior. In our previous study, we found that transitioning offspring from HF fed dams to a control diet at weaning lessens, but does not ameliorate, metabolic disturbances (Page et al., 2009). These results are consistent with other studies showing that transitioning pups from HF fed dams to control chow at weaning does not fully compensate for maternal diet effects associated with the significant increase in leptin and anxiogenic outcomes in the adult offspring (Bilbo and Tsang, 2010). In our current study, the excessive traveling recorded for our animals in the closed arms of the EPM and the significant reduction in open-arm time indicate an exacerbation of anxiety-like behavior in response to both the maternal and post-weaning HF diet. Our findings also agree with results from studies which demonstrate that non-human primates exposed to a maternal or post-weaning HF diet exhibit a persistent increase in anxiety and cortisol levels (Thompson et al., 2017); and that maternal hyperleptinemia and leptin resistance resulting from diet-induced obesity during pregnancy have been linked to anxiety-like behaviors in non-human primate offspring (Sullivan et al., 2010) and mice (Peleg-Raibstein et al., 2012). A recent study in rats also shows that maternal obesity and hyperleptinemia are associated with an impairment in stress adaptation and anxiety-like behavior in male offspring (Balsevich et al., 2016).

The dramatic increase in circulating leptin following exposure to a maternal and/or post-weaning HF diet suggests that leptin resistance develops in animals consuming a HF diet (Page et al., 2009; Vasselli et al., 2013). Studies also indicate that obesity and disturbances in leptin signaling markedly affect the expression of neuronal and glial proteins (Ahima et al., 1999). Disturbances in leptin signaling are particularly important in the hippocampus. The high level of hippocampal leptin receptors (Caron et al., 2010) promotes neurogenesis and efficient synaptic transmission following leptin activation (Grillo et al., 2011; McGregor and Harvey, 2018), and leptin binding stimulates rapid changes in dendritic morphology (O’Malley et al., 2007). In fact, Ahima et al. (1999) showed that rats with either a leptin deficiency or insensitivity had reduced brain weights and protein content and that the levels of several synaptic proteins were significantly lower compared to control animals, in particular, syntaxin, synaptobrevin, and SNAP25.

Our results support the claim that excessive fat consumption is associated with a disturbance in synaptic efficacy. Following exposure to maternal and post-weaning HF diets, we detected a significant reduction in both mRNA and protein levels for SNAP25 and synaptophysin, two proteins critical for the proper functioning of presynaptic vesicle release, as well as for MAP2, a key dendritic marker. Our data are also consistent with studies claiming that a reduction in synaptic spines and dendritic remodeling are prominent characteristics in animal models of depression (Duman and Duman, 2015). A significant reduction in SNAP-25 expression is also associated with impaired dendritic spine formation and function (Tomasoni et al., 2013) as well as neuronal circuit disruption and altered plasticity (Antonucci et al., 2013). We previously reported that mRNA levels of synaptophysin decrease in response to high-fat feeding and hyperleptinemia (Page et al., 2014), and in this study we confirmed that a significant reduction in synaptophysin’s protein level also occurs. Diminished expression of SNAP25 and synaptophysin in our animals is particularly interesting, since studies have shown that these two vesicle membrane proteins play a critical role in plasticity as well as associated cognitive and behavioral functions (Schmitt et al., 2009). For example, the marked reduction in SNAP-25 expression in our study is associated with increased immobility during the FST and reduced open arm time during EPM testing. Our studies support results generated from SNAP-25 knock-out mice which show that these mice exhibit a dramatic increase in body weight, adiposity, hyperleptinemia, leptin resistance and impaired stress-coping behavior (Valladolid-Acebes et al., 2015).

Brain effector molecules, such as GDNF, also exert regulatory influence in the CNS. In fact, GDNF has been shown to promote neuronal development and differentiation and to play an important neuroprotective role during neuronal and glial cell maturation, growth, and survival (Tsybko et al., 2017). Moreover, severe mood disorders such as MDD and bipolar affective disorder have been characterized by dysregulation of neurotrophic signaling. Both GDNF and leptin exert neurotrophin-like actions in the hippocampus (Mainardi et al., 2017), and disturbances in the availability and function of either effector has been implicated in the development of mood disorders. Recent evidence supports the hypothesis that GDNF is critical to hippocampal dendrite development and results from *in vitro* and *in vivo* experiments show that GDNF and its receptor are required for the proper growth and morphology of dendritic arbors and spines (Irala et al., 2016). Reduced GDNF mRNA and serum levels have been detected in patients with major depression (Otsuki et al., 2008) and serum GDNF level was negatively correlated with the severity of depressive symptoms. Conversely, an increase in GDNF has been shown to act as a neuroprotectant which prevents oxidative injury in neural cells (Chen et al., 2005; Ortiz-Ortiz et al., 2011). Our study indicates that GDNF expression is significantly reduced following exposure to excess saturated fat in the maternal and/or post-weaning diet. These findings confirm that early-life programming persists into adulthood and that continuous exposure to excess saturated fat during early life and adulthood intensifies the adverse effect on an animal’s emotional balance.

As noted above, our results indicate that significant reductions in the expression of key proteins mediating synaptic function are also associated with decreased stress coping strategies and increased anxiety-like behavior in animals exposed to HF diets during gestation, lactation and/or post-weaning. We investigated these disturbances further by examining the interdependent relationship among neurotrophins, serotonin receptors, and emotionality. Both animal and human studies have implicated abnormal serotonergic function in the pathophysiology of anxiety and depression (Zmudzka et al., 2018). Our results provide further evidence for serotonergic dysfunction since a significant reduction in expression of the serotonin 5HT1A receptor was detected in animals exposed to maternal and/or post-weaning HF diets. In contrast, 5HT2A receptor expression was increased only in response to the maternal HF diet. The relationship between the reduced expression of both GDNF and serotonin 5HT1A receptor in the hippocampus may, in part, be due to GDNF’s role in the survival and maintenance of monoaminergic neuronal function (Pascual et al., 2011) and, in turn, hippocampal 5-HT1A receptor’s mediation of GDNF’s antidepressant effects (Naumenko et al., 2013). Disturbances in the 5HT1A and 5HT2A receptors have been implicated in the development of stress-related illness such as major depressive disorder (MDD) (Stockmeier, 2003; Noro et al., 2010), and enhancing 5-HT1A receptor gene expression and activity predicts a favorable outcome for anti-depressant action (Albert and Francois, 2010).

Additional studies have demonstrated a more defined link between 5HT1A, 5HT2A, and emotional disturbances. For example, increased 5HT2A receptor binding is associated with depressed mood, impulsivity, and suicidality (Bilbo and Tsang, 2010); whereas, developmental deficiencies in 5HT1A receptor expression increase an individual’s susceptibility to anxiety disorders (Donaldson et al., 2014). Interestingly, a recent report suggests that a reciprocal competitive interaction exists between the two receptors. Data from this study indicates that inhibition of 5HT1A receptor expression results in a significant increase in 5HT2A receptor protein. Conversely, activation of 5HT2A receptors suppresses the expression of 5HT1A receptor and promotes anxiety-like behavior (Xiang et al., 2017, 2019). Our data provide further evidence to support the hypothesis that serotonin receptors 5HT1A and 5HT2A are engaged in a competitive interaction which is associated with anxiety-like behavior and affective disorders.

To further explore the possibility that exposure to excess saturated fat during development is involved in the impairment of synaptic functions and emotional stability, we analyzed the expression of two key subunits of glutamate’s AMPA receptor, GluA1 and GluA2. These subunits are critical for glutamate’s effective interaction with the AMPA receptor and, in concert with additional neurotrophic factors, they regulate developmental and adult neuroplasticity (Duman, 2014b). Evidence suggests that, in addition to changes in serotonergic function, disturbances in glutamatergic neurotransmission may be central to mood disorders (Hashimoto, 2011; Tokita et al., 2012; Hashimoto et al., 2013). Loss of coping ability and depression have been associated with compromised AMPA receptor activation due, in part, to changes in expression of the GluA1 subunit (Duric and Duman, 2013); whereas, enhanced activation of AMPA receptors promotes an antidepressant effect (Farley et al., 2010). In fact, AMPA-receptors containing the GluA1 subunit are required for the antidepressant action of NMDA antagonists (Koike and Chaki, 2014). Our data extend support for these findings. The significant reduction in expression of the AMPA receptor GluA1 subunit in our obese animals suggests that hippocampal glutamatergic signaling is compromised by high-fat feeding and is accompanied by increased emotionality.

Together, our results show that exposure to a diet high in saturated fat during critical stages of brain development disturbs the expression of hippocampal genes involved in synaptic function and predisposes the animal to mood disorders. We detected a marked reduction in GDNF and significant decreases in the expression of genes coding for markers of synaptic function, MAP2, SNAP-25, synaptophysin, key serotonin receptors, and critical subunits of the glutamate AMPA receptor. These changes were associated with a reduction in the animal’s stress-coping strategy and increased anxiety-like behaviors.

It should be noted that behavioral profiles are not directly dependent on a selection of molecular markers, rather they are a manifestation of complex molecular integrations and environmental contexts. For example, dysfunction in the central serotonergic system is prominently positioned in the pathoetiology of depression, and yet, its role in this behavioral manifestation has not been clearly elucidated. This also pertains to high-fat diet-induced neurological and cognitive dysfunctions due to neural inflammation, oxidative stress or decreased neurogenesis and plasticity in the hippocampus. In our study, we examined the expression of a few representative proteins that are likely to contribute to hippocampal plasticity changes and associated behavioral manifestations. Ultimately the results from our study and a multitude of others will provide a foundation for a more mechanistic hypothesis which will be derived from a combination of factors.

In summary, our results suggest that the levels of hippocampal proteins required for synaptic function are inadequate in rat offspring exposed to either a maternal or post-weaning high-fat diet. Moreover, exposure to the combination of maternal and post-weaning HF diets increases the magnitude of adverse consequences. Impaired neural functions resulting from the physiological stress of a diet high in saturated fat are likely to underpin significant disturbances in emotional balance.

## Data Availability Statement

All datasets generated for this study are included in the article/supplementary material.

## Ethics Statement

The animal study was reviewed and approved by Bucknell University Animal Care and Use Committee.

## Author Contributions

KP performed the experiments and analyzed the data. KP wrote the manuscript with contributions from EA. Both authors conceived and designed the project, discussed the data, and contributed to the article and approved the submitted version.

## Conflict of Interest

The authors declare that the research was conducted in the absence of any commercial or financial relationships that could be construed as a potential conflict of interest.

## Footnotes

1 http://www.ncbi.nlm.nih.gov

2 https://blast.ncbi.nlm.nih.gov/Blast.cgi
